# A Case of Camptocormia (Bent Spine) Secondary to Early Motor Neuron Disease

**DOI:** 10.1155/2004/163957

**Published:** 2004-06-09

**Authors:** Feriha Ozer, Aytul Mutlu, Hasan Meral

**Affiliations:** Department of NeurologyHaseki Research and Educational HospitalIstanbulTurkey

**Keywords:** camptocormia, motor neuron disease, Parkinson's disease, segmental dystonia, muscle biopsy

## Abstract

Camptocormia is a gait disorder, characterized by hyperflexion of thoracolumbar spine which increases on walking, and disappears in the supine position.

A 48 year-old man developed progressive gait deterioration for one year and slight weakness and tremor of both hands for five months. It eventually became apparent that the patient had motor neuron disease, as well as symptoms of extrapyramidal disorder.

## 1. Introduction

Camptocormia is a gait disorder characterized by flexion of thoracolumbar spine which appears and gradually increases with walking and disappears in the supine position. This phenomenon was first described by Brodie in 1837. During the First World War, it was commonly seen in young soldiers and attributed to a psychogenic conversion reaction [11]. This phenomenon has now been associated with wide spectrum of organic disorders. Primary forms are due to segmental dystonia of the abdominal wall muscles [10] and secondary forms to tumors of the spine, infections, intradural hematoma, ankylosing spondylitis, spinal stenosis [2,11], some forms of muscular dystrophies involving paraspinal abdominal muscles [6], myopathies [4, 7,9,12,13,15], a side effect of valproic acid [4], and neurogenic disorders such as amyotrophic lateral sclerosis [17], and Parkinson's disease [1,3,16,18].

A few cases as a manifestation of the late stages of amyotrophic lateral sclerosis (have also been reported), but the case reports of camptocormia as a manifestation of the early stages of amyotrophic lateral sclerosis are extremely rare. There was only one case report. Van Gerpen reported camptocormia in a patient in the early stages of amyotrophic lateral sclerosis [17].

We describe a patient with camptocormia secondary to the early stages of motor neuron disease, who also had some symptoms of parkinsonism.

## 2. Case report

A 48 year-old man developed progressive gait deterioration for one year with slight weakness and tremor of both hands for five months.

The patient was cognitively unimpaired. He had a severe antiflexion of thoracolumbar spine, particularly increased by walking, which completely disappeared while sitting and in the supine position. At the onset of walking, the patient could sustain the erect posture by supporting himself with his hands on his thighs. After a few minutes of walking the spine gradually flexed. He had some pelvic instability while walking in association with proximal weakness of the legs. He had an asymmetric arm posture during walking with extension of the shoulder on the right and flexion of the elbow on the left side (Fig. 1(A–B)).

The patient had slight parkinsonism manifested by a facial hypomimia, diminished stride length and arm swing on the right side, mild cogwheel rigidity of right wrist and bilateral postural tremor. Diffuse fasciculations were evident at the shoulder and pelvic girdle

ISSN 0953-4180/04/$17.00 © 2004 – IOS Press and the authors. All rights reserved

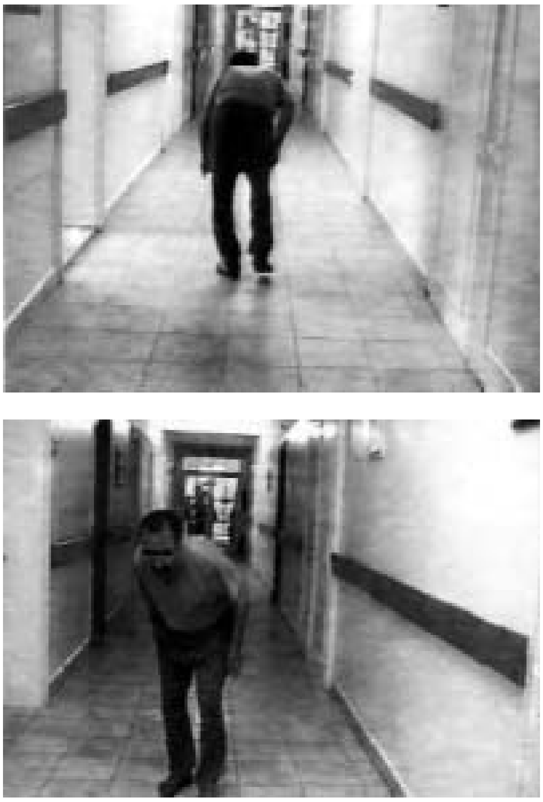

A)

B)

Fig. 1. Patient with camptocormia showing antiflexion of trunk, extention of right arm A) Posterior appearance B) Anterior appearance.

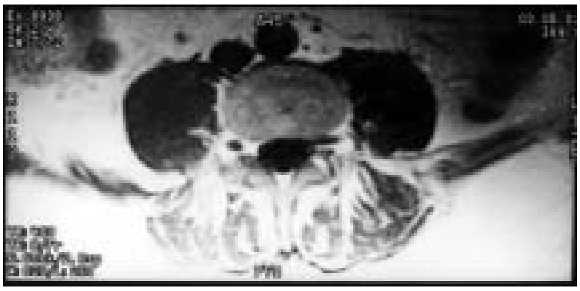

Fig. 2. On T1-weighted images, a significant volume loss of deep paravertebral dorsal muscles and remarkable hyperintensity implicating fatty.

muscles bilaterally. Strength was reduced in the axial muscles and finger extensor muscles bilaterally. There was no involvement of the other limb muscles, neck flexors, extensors and bulbar muscles. There was a mild atrophy of the interosseous hand muscles thnar and hypothenar muscles. Muscle stretch reflexes were diminished. Plantar reflexes were flexor.

There was no sensory deficit. There was no contributory family, history no autonomic, urinary or cerebellar dysfunction. A short test of mental status was 36/38 points (described by Kökmen E. 1987).

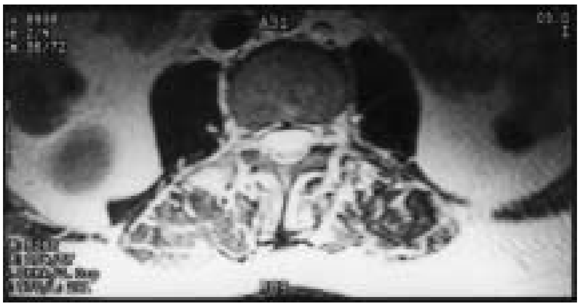

Fig. 3. Axial T2- weighted images: Diffuse symmetric signal intensity.

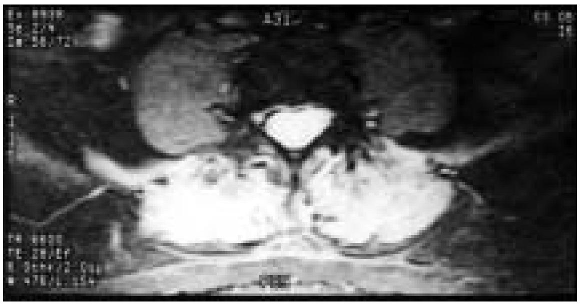

Fig. 4. Axial T2- FSE MRG: Diffuse symmetric signal intensity.

Electromyography (EMG) demonstrated fasciculation and fibrillation potentials at rest and polyphasic motor unit potentials with long duration and high amplitude (giant) on voluntary contraction with discrete recruitment pattern in three limb muscles (Right Deltoid, Biceps, APD, ADM, right and left RF, TA, Gastrocneimous, EDB, AH), and paravertebral lumbar muscles. Nerve conduction studies were within normal limits except for bilateral low amplitude median compound muscle action potentials [RG4].(wrist: 0.5 mV; elbow: 0.4 mV).

In laboratory investigations, blood count, erythrocyte sedimentation rate, liver function tests, serum electrolytes, serum vitamin B-12 level, paraneoplastic antgibody profile, thyroid hormones, antinuclear antibodies, human immunodeficiency virus titres and syphilis tests were in normal limits. Level of CPK was slightly elevated at 305 U/L(26–174 U/L). Cervical, thoracal, lumbar direct graphies and thoracal and abdominal CT findings were normal. Dorsal spinal MRI showed mild spondylosis. On T1-weighted images, significant volume loss of paravertebral dorsal muscles and pathological hyperintensity consistent with fatty degeneration were apparent (Figs 2, 3 and 4). Cranial MRI imaging was normal.

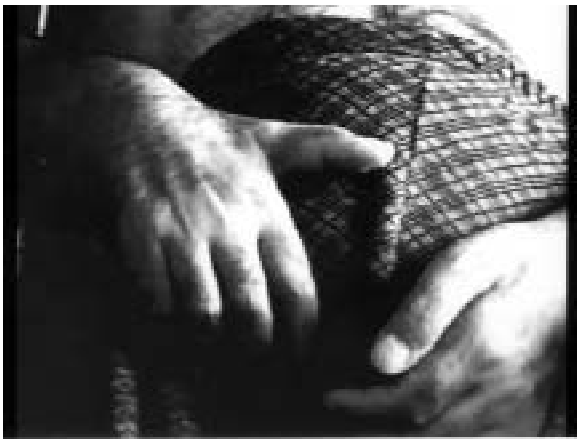

Fig. 5. Atrophy at the interosseous hand muscles.

Muscle biopsy specimen was taken form the erector spinae muscles at level T 11. Cross- sectioned muscle fibers showed considerable variation in fiber diameter with atrophic and hypertrophic fibers. Atrophic fibers was including pycnotic nuclear clumps. There was no necrosis, phagositosis, endomysial or perimysial inflammatory infiltrate and regeneration. Most of the muscle fibers were of target cell appearance.

Three months later his gait had deteriorated with weakness of the axial, limb and girdle muscles. There was severe atrophy of the interosseous hand muscles, thenar and hypothenar muscles (Fig. 5). Muscle stretch reflexes were diminished. After one year, the neurological examination showed further deterioration. He had weakness of the cervical flexor and extansor muscles, weakness and severe wasting of thoracic paraspinal and distal leg muscle. He had dysphonia and slurred speech. He could not stand or walk because of weakness. There was no autonomic dysfunction. Intellectual function was still normal and there were no hallucinations. Cranial and cervical MRI was normal. Two months later he developed weakness of the respiratory muscles and dysphagia and died of respiratory failure.

## 3. Discussion

This patient's pronounced forward flexion of the trunk was a feature of a lower motor neuron disorder and related to weakness of paraspinal muscles. EMG suggested a neurogenic denervation of the paraspinal muscles that was confirmed by biopsy to be neurogenic. MRI scans revealed variable degrees of atrophy and fatty degeneration of thoracolumbar paraspinal muscles.

Dijaldetti et al. reported eight patients with PD that manifested camptocormia, and implied that camptocormia could be central in origin [3]. Charpentier et al. reported camptocormia in a patient with Parkinson's disease and suggested camptocormia could be due to myositis [1]. Camptocormia has been reported at the late stages of amyotrophic lateral sclerosis. Kuncl et al reported a high frequency of truncal muscle involvement in amyotrophic lateral sclerosis, and emphasized its importance as a diagnostic clue [5]. Camptocormia is extremely rare at the early stages of amyotrophic lateral sclerosis. Van Gerpen reported camptocormia in a patient at the early stages of amyotrophic lateral sclerosis [17] who held his arms behind him when he was walking. There are reports in the literature of patients with Parkinson's disease and camptocormia who hold their arms immobile on their sides or slightly anteriorly. In this case, the patient's right arm was held extended at the shoulder similar to the patient described by Van Gerpen implicating the presence of the motor neuron disease or other neuromuscular diseases. However, the patient also presented some signs of parkinsonism, including hypomimia, bradykinesia and diminished arm swing on the right side.

According to the Multiple System Atrophy (MSA) consensus criteria, for a diagnosis of probable MSA, parkinsonism and cerebellar findings must be associated with autonomic and urinary dysfunction. MSA was not diagnosed because there was no autonomic, urinary or cerebellar findings and Lewy Body disease was not diagnosed because of normal cognitive tests. Camptocormia has been described as an early feature of idiopathic Parkinson's disease. Its Dopa responsiveness is poor. Although this patient's camptocormia might have been secondary to IPD or both motor neuron disease and PD, such a severe gait abnormality would be an unusual feature of the early stages of PD. In our patient, the extrapyramidal features were stable, but the camptocormia progressed in parallel with muscle weakness and atrophy. Atrophy and fasciculations were wide-spread. For this reason, it was concluded that camptocormia was due to motor neuron disease and findings of parkinsonism was coincidental.

Perticoni et al reported 11 sporadic form of parkinsonism associated with motor neuron disease. Combined lesions of upper and lower neuron were found in 10 of 11 cases, while in one case an isolated lower motor neuron disorder was present as in our patient. In 6 patients, both syndromes manifested at the same time [8].

Van Gerpen reported another camptocormia case. In that case, he concluded that the postural abnormality was compatible with an axial, task-specific dystonia and was levodopa-responsive [19].

In conclusion, this patient's camptocormia was attributable to involvement of paraspinal muscles in the early stage of motor neuron disease and antedated the classical findings of motor neuron disease.
